# Changes in Ultrasound-Based Risk Stratification and Surgical Selection of Adnexal Masses During the COVID-19 Pandemic: A Single-Center Retrospective Study

**DOI:** 10.3390/diagnostics16172719

**Published:** 2026-08-26

**Authors:** Balazs Erdodi, Gergo Jozsef Szollosi, Fanni Torok, Laszlo Varadi, Zoard Tibor Krasznai, Attila Jakab

**Affiliations:** 1 Department of Obstetrics and Gynecology, Faculty of Medicine, University of Debrecen, 4032 Debrecen, Hungary; torok.fanni@med.unideb.hu (F.T.); krasznai.zoard@med.unideb.hu (Z.T.K.); ja@med.unideb.hu (A.J.); 2 Doctoral School of Clinical Sciences, University of Debrecen, 4032 Debrecen, Hungary; 3 Coordination Center for Research in Social Sciences, Faculty of Economics and Business, University of Debrecen, 4032 Debrecen, Hungary; szollosi.gergo@econ.unideb.hu; 4 Szent Margit Hospital, 1032 Budapest, Hungary; dr.varadi.laszlo@sztmargit.hu

**Keywords:** COVID-19, adnexal mass, gynecologic ultrasound, IOTA, ADNEX model, risk stratification, ovarian neoplasm

## Abstract

**Background/Objectives:** The COVID-19 pandemic disrupted gynecologic care pathways and required stricter prioritization of surgical treatment. We evaluated whether the pandemic restriction period was associated with changes in the characteristics of women with adnexal masses reaching surgery and explored ADNEX discrimination within the histologically verified surgical cohort. **Methods:** This retrospective single-center study included all consecutive women evaluated for an adnexal mass in a dedicated gynecologic ultrasound clinic during a pre-pandemic baseline period and a COVID-19 restriction period. Ultrasound examinations were performed by the same expert using International Ovarian Tumor Analysis (IOTA) terminology and the IOTA Assessment of Different NEoplasias in the adneXa (ADNEX) score was calculated prospectively at the time of examination. Histological analyses were restricted to surgically managed patients. **Results:** Overall, 340 women were evaluated in the non-COVID period and 211 during the COVID period; 149 (43.8%) and 40 (19.0%), respectively, underwent surgery. After exclusion of five adolescents from the pre-pandemic group, 144 and 40 operated patients were analyzed. The COVID-era surgical cohort was older (median 52.0 vs. 39.0 years, *p* = 0.0053), had higher ADNEX scores (median 13.7% vs. 4.4%, *p* = 0.0010), and had a higher proportion of borderline or malignant histology (32.5% vs. 13.2%, *p* = 0.0083). In an exploratory pooled logistic model, ADNEX score and COVID-period status were associated with non-benign histology. The AUCs were 0.789 and 0.905 in the COVID and non-COVID surgical cohorts, respectively, without a statistically significant between-cohort difference (*p* = 0.169). **Conclusions:** The COVID-19 restriction period was associated with a lower proportion of evaluated women proceeding to surgery and with enrichment of the operated cohort for older patients and lesions with higher sonographic risk and non-benign histology. Because surgical selection itself depended partly on ultrasound findings and outcomes were unavailable for non-operated women, these data should not be interpreted as independent validation of ADNEX or proof that deferred management was safe.

## 1. Introduction

The COVID-19 pandemic had a profound and tragic impact on healthcare systems worldwide. As large numbers of infected patients required hospitalization, many surgical procedures were canceled or postponed, while intensive care units were occupied by critically ill respiratory cases [1,2]. To reduce transmission, governments introduced lockdowns and self-isolation measures on an unprecedented scale [3]. These changes affected not only treatment pathways but also diagnostic workflows, as healthcare systems had to adapt to reduced capacity and shifting priorities. In addition, many patients postponed follow-up visits or diagnostic evaluations because of movement restrictions or fear of infection [4]. Delays in cancer diagnosis are clinically important because they may adversely affect treatment response and overall survival. Increased mortality related to pandemic-associated delays in cancer care has been reported in both the United States and the United Kingdom [5,6]. Early diagnosis is particularly important in ovarian cancer, which often presents with nonspecific symptoms and may therefore remain undetected until a more advanced stage [7].

Ultrasonography, especially transvaginal ultrasound, plays a central role in the assessment of pelvic adnexal masses. Its main advantages are wide availability, low cost, and high diagnostic performance. Depending on the experience of the examiner, ultrasound evaluation can be further supported by validated mathematical models to improve diagnostic accuracy. Over the past two decades, the International Ovarian Tumor Analysis (IOTA) group has developed several evidence-based tools for the characterization of adnexal lesions. The IOTA Simple Rules can be used by less experienced sonographers and have shown high sensitivity and specificity, approaching the performance of expert pattern recognition [8]. When a more precise numerical estimate of malignancy risk is needed, the Simple Rules Risk model can be applied [9]. The most advanced logistic regression model developed by the IOTA group is the Assessment of Different NEoplasias in the adneXa (ADNEX) model. It incorporates three clinical variables (patient age, oncology center status, and CA125 value) together with six ultrasound variables (maximum lesion diameter, maximum diameter of the solid component, presence of more than 10 locules, acoustic shadows, number of papillary projections, and ascites) [10]. The model can be used irrespective of the examiner’s subjective level of expertise. It may also be applied without CA125, although omission of the marker reduces the precision of subclassification among borderline, stage I, stage II–IV, and metastatic tumors [10]. When used to discriminate between benign and malignant disease, the ADNEX model has demonstrated excellent diagnostic performance, with an AUC of 0.920 (95% CI 0.905–0.934), although sensitivity and specificity depend on the chosen threshold [11].

The management of adnexal masses became especially challenging during the pandemic because these lesions range from clearly benign cysts to borderline tumors and invasive malignancies, while often presenting with overlapping symptoms and imaging appearances. Under conditions of restricted operating-room access and delayed referrals, reliable preoperative triage became increasingly important. Standardized sonographic assessment and validated prediction tools such as the IOTA ADNEX model offered a structured approach to malignancy-risk estimation. The novelty of the present study is not the validation of a new diagnostic model, but the real-world evaluation of how the case mix of adnexal masses reaching surgery changed when an established ultrasound-based risk-stratification framework was used under unusually constrained healthcare conditions.

Therefore, the aim of this study was to compare the characteristics, ultrasound findings, ADNEX risk estimates, and histological outcomes of adnexal masses reaching surgery before and during the COVID-19 restriction period in a tertiary referral center. A secondary, exploratory aim was to describe the discriminatory performance of ADNEX within the surgically selected, histologically verified cohorts.

## 2. Materials and Methods

This retrospective observational study was conducted at the Department of Obstetrics and Gynecology, University of Debrecen, Debrecen, Hungary. All consecutive women in whom an adnexal mass was identified in the dedicated gynecologic ultrasound outpatient clinic during the predefined study intervals were entered into the clinical database; no additional selection criteria were applied at the initial ultrasound assessment. The non-COVID reference interval extended from 30 March 2017 to 7 March 2019 and was deliberately chosen to provide a sufficiently large and stable pre-pandemic baseline. The COVID interval extended from 1 September 2020 to 1 March 2021 and represented a period of major local restrictions affecting elective surgical activity. Because the observation periods differed substantially in duration, examination and operation rates per month are reported and the comparisons are interpreted as differences in case mix rather than as a direct comparison of absolute service volumes.

All ultrasound examinations were performed by the same IOTA-certified expert examiner (B.E.), who had more than 15 years of experience in gynecologic ultrasonography. A Samsung HERA W10 high-end ultrasound system (Samsung Medison, Seoul, Republic of Korea) was used throughout the study period with an EV3-10B transvaginal probe and, when required, a CV1-8A transabdominal probe. Examinations were performed according to IOTA terms and definitions [12]. Recorded variables included lesion size, IOTA tumor morphology, color score, acoustic shadows, papillary projections, ascites, and other components required for ADNEX calculation.

The ADNEX score was calculated prospectively at the time of the ultrasound examination using the integrated ADNEX application available on the ultrasound system. The calculated score was visible to the examiner and was therefore not blinded during clinical decision-making. CA125 was incorporated when available (119/144 [82.6%] non-COVID patients and 31/40 [77.5%] COVID patients); when CA125 was unavailable, the validated ADNEX version without CA125 was used [10]. This approach reflects routine clinical practice, in which ADNEX can be applied with or without CA125. The decision to operate was not determined by ADNEX alone, but was based on the complete ultrasound report, expert pattern recognition, symptoms and clinical context and the opinion of the treating gynecologist/gynecologic oncologist.

**Figure 1:**
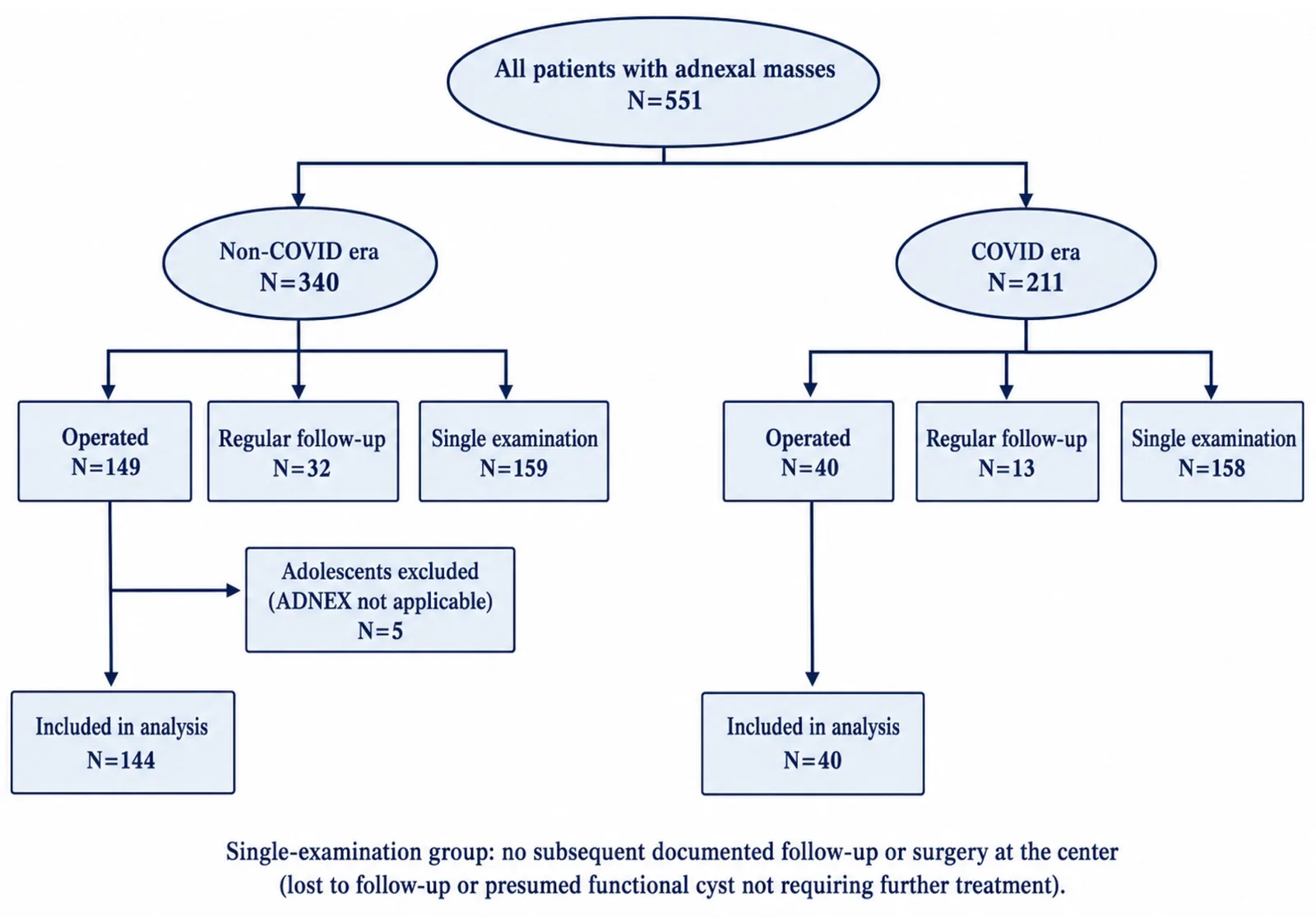
Patient data flowchart—The single-examination group had no subsequent documented follow-up or surgery at the study center and included women lost to follow-up and women with presumed functional cysts not requiring further treatment.

The primary unit of analysis was the patient. In women with bilateral or multiple adnexal masses, the index lesion was defined prospectively as the most complex mass in accordance with IOTA recommendations; the ADNEX score and the corresponding histological outcome were assigned to this index lesion. Five adolescents younger than 14 years in the pre-pandemic operated group were excluded because ADNEX is not applicable in this age group. Women managed conservatively without histological verification were not included in analyses using histology as the reference standard. The patients shown in Figure 1 as having a single examination had no subsequent documented follow-up or surgery at our center; this group comprised women lost to follow-up and women with presumed functional cysts for which no further treatment was considered necessary. For descriptive lesion-morphology summaries, all recorded lesions were retained and lesion-level denominators are explicitly reported. Color score was summarized at patient level using the highest recorded score among multiple lesions, and acoustic shadowing was considered present if present in any recorded lesion.

Histopathology served as the reference standard for operated patients. Diagnoses were extracted from the original routine pathology reports; no centralized retrospective slide review was performed. Approximately three to four staff pathologists were responsible for gynecologic specimens during the study periods, and the same departmental WHO-based diagnostic framework and routine criteria were applied in both eras. Histological diagnoses were grouped as benign, borderline or malignant for the primary analyses. In bilateral cases, the pathology assigned to the index lesion used for ADNEX was used as the patient-level outcome. Tumor grade and FIGO stage were not systematically captured in the study database and were therefore not analyzed. The normalized histopathological spectrum derived from the original reports is provided in Supplementary Table S1.

Statistical analyses were performed using the patient as the primary analytical unit. Normality of continuous variables was assessed using the Shapiro–Wilk test. Because age, ADNEX score and maximum lesion diameter were non-normally distributed, they are reported as medians and interquartile ranges (IQRs) and compared using the Mann–Whitney U-test; rank-biserial correlation is provided as an effect-size measure. Categorical variables were compared using Fisher’s exact test or the chi-square test, as appropriate. The pooled multivariable logistic regression model included ADNEX score and study period as predictors of borderline/malignant versus benign histology; age was not entered separately because it is already a component of the ADNEX model. The model included 184 patients and 32 non-benign outcomes and odds ratios (ORs) are reported with 95% confidence intervals (CIs). No imputation was performed; analyses used available data. ROC analyses were considered exploratory because histological verification was available only for surgically selected patients. AUCs were estimated with 95% CIs and compared using DeLong variance estimates for independent cohorts. Youden-derived thresholds were reported descriptively with exact binomial 95% CIs for sensitivity and specificity. A two-sided *p*-value <0.05 was considered statistically significant.

## 3. Results

A total of 551 women were evaluated across the two study periods (non-COVID: n = 340; COVID: n = 211). The pre-pandemic interval comprised 23.3 months and the COVID restriction interval 6.0 months. This corresponded to approximately 14.6 versus 35.3 examinations per month and 6.4 versus 6.7 operations per month, respectively. However, the proportion of evaluated women proceeding to surgery was substantially lower during the COVID period (40/211, 19.0%) than during the non-COVID period (149/340, 43.8%; Fisher’s exact *p* = 1.4 × 10^−9^). Five adolescents were excluded from the pre-pandemic operated group, leaving 144 non-COVID and 40 COVID patients for the histology-based analyses. In addition, 32 and 13 patients, respectively, underwent documented regular follow-up, whereas 159 non-COVID and 158 COVID-era patients had only a single examination without subsequent documented follow-up or surgery at our center (Figure 1).

The operated COVID-era cohort was significantly older and had higher ADNEX risk estimates than the operated non-COVID cohort (Table 1). Median age increased from 39.0 to 52.0 years (*p* = 0.0053), and median ADNEX risk increased from 4.4% to 13.7% (*p* = 0.0010). In contrast, the maximum lesion diameter did not differ significantly between the groups (median 66.0 vs. 74.0 mm, *p* = 0.1452), suggesting that the change in the surgically treated case mix was more closely related to overall risk characteristics than to lesion size alone.

At patient level, the distribution of maximum color scores differed between periods (*p* = 0.00025), with a higher proportion of score-4 vascularity in the COVID cohort. Acoustic shadowing was more frequent in the COVID cohort (60.0% vs. 43.8%), although this difference was not statistically significant (*p* = 0.0758). At lesion level, multilocular-solid and solid morphology represented 55.5% of recorded COVID-era lesions compared with 30.2% of non-COVID lesions; because bilateral lesions were not statistically independent, these morphology data are presented descriptively without an inferential between-group test. Details are shown in Table 2.

**Figure 2:**
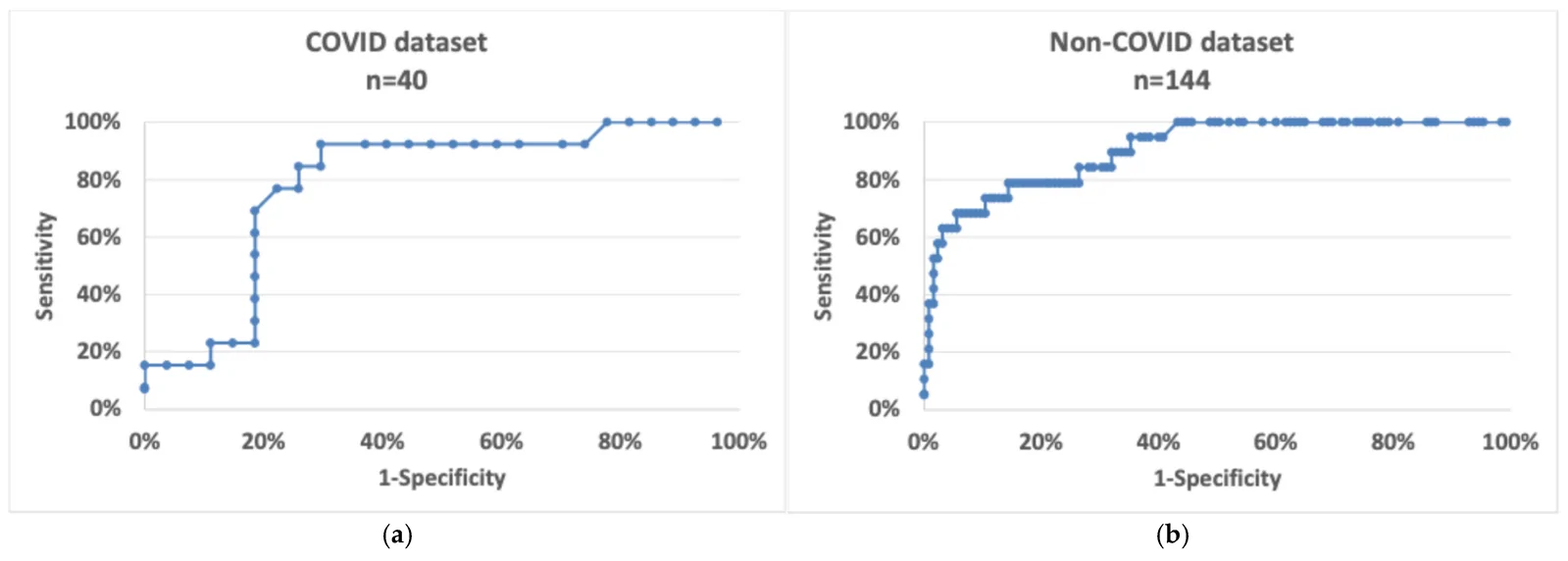
Exploratory ROC analysis of ADNEX within the surgically selected cohorts: (**a**) COVID dataset, AUC 0.789 (95% CI 0.637–0.941); (**b**) non-COVID dataset, AUC 0.905 (95% CI 0.839–0.972).

Exploratory ROC analysis (Figure 2) yielded an AUC of 0.789 (95% CI 0.637–0.941) in the COVID cohort and 0.905 (95% CI 0.839–0.972) in the non-COVID cohort. The difference between the two independent AUCs was not statistically significant (*p* = 0.169). The Youden-derived threshold was 15.8% in the COVID cohort, with sensitivity 92.3% (95% CI 64.0–99.8%) and specificity 70.4% (95% CI 49.8–86.2%); the corresponding PPV and NPV were 60.0% and 95.0%. In the non-COVID cohort, the exploratory threshold was 25.1%, with sensitivity 78.9% (95% CI 54.4–93.9%) and specificity 85.6% (95% CI 78.2–91.2%); PPV and NPV were 45.5% and 96.4%, respectively. Because these thresholds were derived retrospectively from already selected surgical cohorts with histological verification, they should not be interpreted as clinical thresholds used for triage or as independent validation of ADNEX in the full outpatient population.

Histopathological outcome confirmed enrichment of the operated COVID-era cohort for non-benign pathology. Borderline or malignant histology was present in 13/40 COVID-era patients (32.5%) compared with 19/144 non-COVID patients (13.2%; Fisher’s exact *p* = 0.0083), corresponding to an OR of 3.17 (95% CI 1.40–7.18). The complete normalized histopathological spectrum derived from the original pathology reports is provided in Supplementary Table S1.

In the pooled exploratory logistic regression model (n = 184; 32 borderline/malignant outcomes), ADNEX risk was associated with non-benign histology (OR 1.045 per 1-percentage-point increase, 95% CI 1.030–1.061; *p* = 3.1 × 10^−9^). After accounting for ADNEX score, COVID-period status was also associated with higher odds of non-benign histology among operated patients (OR 2.87, 95% CI 1.06–7.78; *p* = 0.0383). Age was not entered separately because it is already incorporated into the ADNEX model. No separate COVID-only regression was performed because the subgroup contained only 13 non-benign events. Summary of the results are shown in Table 3.

**Figure 3:**
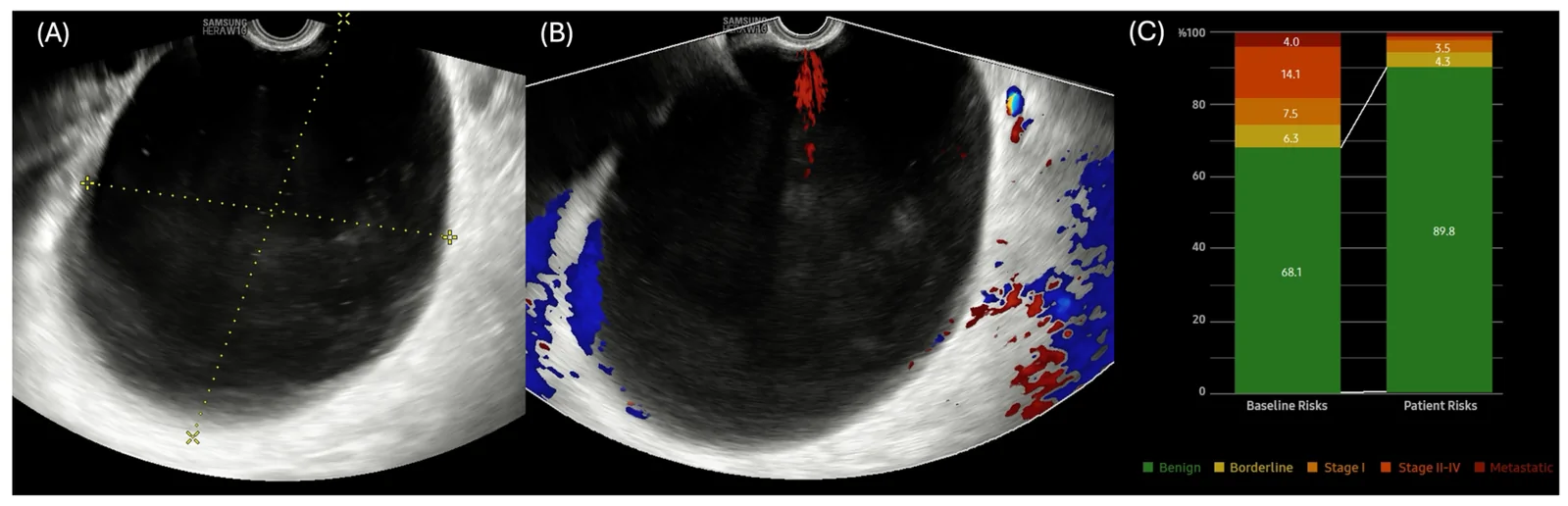
Representative adnexal mass with low-risk ultrasound features. (**A**) Transvaginal grayscale ultrasound image demonstrating a large, unilocular cystic adnexal lesion with smooth walls and no obvious solid component or papillary projection. (**B**) Color Doppler image showing no relevant internal vascularization, with flow signals limited to the peripheral tissues. (**C**) Corresponding IOTA ADNEX risk calculation indicating a predominantly benign risk profile. Final histology confirmed benign disease (histology: serous cystadenoma).

**Figure 4:**
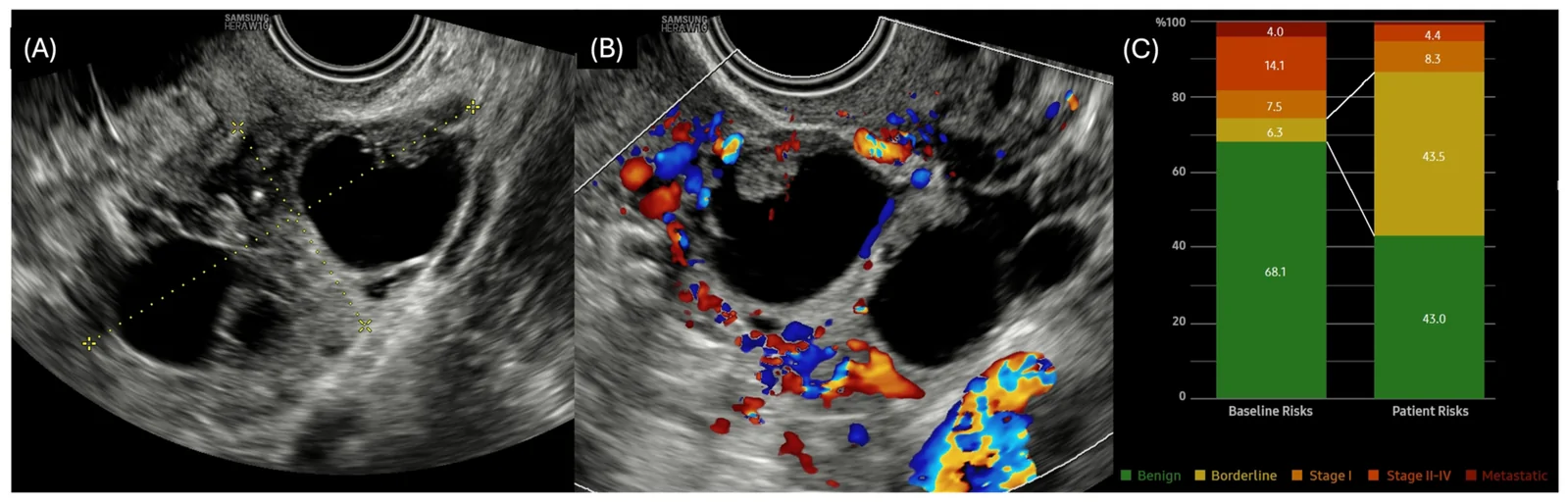
Representative adnexal mass with borderline characteristics. (**A**) Transvaginal grayscale ultrasound image demonstrating a multilocular-solid cystic adnexal lesion with septations, papillary projections and increased morphologic complexity compared with the benign representative case. (**B**) Color Doppler ultrasound image showing vascularization within the lesion also in the papillary projections and septal structures, supporting a more suspicious sonographic appearance. (**C**) Corresponding IOTA ADNEX risk calculation demonstrating increased risk of non-benign disease, particularly in the borderline tumor category showing an increased predicted risk in the borderline tumor subgroup. Final histology confirmed a borderline ovarian tumor (histology: mucinous borderline ovarian tumor).

Representative ultrasound images illustrating the range of adnexal morphology and corresponding ADNEX risk estimates are presented in Figure 3 and Figure 4.

## 4. Discussion

The main finding of this study is that the COVID-19 restriction period was associated with a marked change in the characteristics of women with adnexal masses who reached surgery. Although the absolute operation rate per month was similar in the two observation windows, the proportion of evaluated women proceeding to surgery fell from 43.8% before the pandemic to 19.0% during the restriction period. The operated COVID-era cohort was older, had higher ADNEX risk estimates, and contained a substantially higher proportion of borderline or malignant pathology. These results therefore describe an enrichment of the surgical case mix rather than demonstrating that a specific diagnostic tool caused the change in selection.

The observed pattern is consistent with the broad reorganization of gynecologic oncology services reported internationally. Frey et al. found that 38.7% of 302 gynecologic oncology patients in New York experienced a COVID-related treatment modification, including modification of 67.4% of planned surgical procedures [13]. In the United Kingdom, Oxley et al. reported a median 50% reduction in urgent gynecologic cancer referrals during the first wave, a 40% median reduction in theater capacity, and postponement of approximately 30% of planned operations [14]. In an ovarian-cancer-specific retrospective cohort from Sao Paulo, Moterani et al. observed a 56.2% reduction in epithelial ovarian cancer diagnoses, an increase in mean time to treatment from 18.9 to 23.0 days, and a lower proportion of patients undergoing surgery during the pandemic [15]. Antunes et al. likewise reported changes in ovarian cancer management, including an increase in the use of neoadjuvant therapy during the period of highest restrictions (55.0% vs. 33.3% before the pandemic and 10.0% during recovery) [16]. Our cohort differs from these studies because it began with all women evaluated for adnexal masses rather than only patients with confirmed gynecologic cancer, thereby providing a view of how the preoperative case mix reaching surgery changed.

The higher ADNEX scores in the COVID-era surgical cohort are notable because maximum lesion diameter did not differ significantly between periods. This suggests that women reaching surgery during restrictions tended to have a higher composite risk profile rather than simply larger masses. However, the present data cannot quantify the independent contribution of ADNEX to the decision to operate. The score was calculated prospectively and was visible to clinicians, but surgical decisions were based on the complete ultrasound report, expert pattern recognition, symptoms, clinical context, and gynecologic-oncology judgment. Consequently, the association between higher ADNEX scores and surgery during the COVID period should not be interpreted causally.

The imaging findings provide a complementary description of this shift in case mix. Maximum color scores were higher during the COVID period, and multilocular-solid or solid morphology was descriptively more common among recorded COVID-era lesions. The illustrative cases in Figure 3 and Figure 4 demonstrate the spectrum from a low-risk benign lesion to a lesion with borderline histology. These observations are consistent with contemporary validation studies showing that standardized IOTA-based approaches and ADNEX provide reproducible risk stratification in routine practice [17,18], while O-RADS similarly emphasizes standardized descriptors and explicit risk categories [19]. Nevertheless, the present study evaluates how these tools were embedded in real-world clinical practice rather than providing a new external validation of either system.

The ROC analyses should therefore be interpreted as exploratory. ADNEX showed an AUC of 0.789 in the COVID surgical cohort and 0.905 in the non-COVID surgical cohort, and the between-cohort difference was not statistically significant. The wider confidence interval in the COVID group reflects its smaller sample and lower number of outcome events. More importantly, histological verification was available only for operated patients, and ultrasound appearance and ADNEX itself could influence whether surgery occurred. The ROC estimates are consequently subject to selection, spectrum, and partial-verification bias. The Youden-derived thresholds of 15.8% and 25.1% were calculated retrospectively from these selected cohorts and must not be interpreted as thresholds that were applied clinically or as evidence that a lower threshold was deliberately adopted during the pandemic.

Histopathology further illustrates the change in the operated case mix: non-benign pathology increased from 13.2% before the pandemic to 32.5% during the COVID restriction period, with a particularly visible increase in borderline tumors. Supplementary Table S1 provides the broader histopathological spectrum, including benign epithelial lesions, endometriosis, germ-cell and stromal tumors, borderline histotypes, primary carcinomas, and metastatic malignancies. Histological diagnoses were taken from routine original reports, and no centralized retrospective slide review was undertaken. Tumor grade and FIGO stage were not systematically captured and therefore cannot be compared between periods. This limits the pathological granularity of the study and prevents conclusions regarding stage migration.

Several alternative explanations should also be considered. The study periods differed in duration and referral patterns may have changed during the pandemic. Patient attendance, decisions to defer benign elective surgery, clinician and patient preferences, and changes in referral pathways could all have contributed to the observed enrichment of higher-risk lesions among operated patients. Furthermore, 159 non-COVID and 158 COVID-era women had only a single examination at our center and had no documented subsequent outcome; some had presumed functional cysts not requiring treatment, whereas others were lost to follow-up. Because outcomes were unavailable for these patients and for conservatively managed masses without histology, the study cannot establish that low-risk lesions were safely deferred, that delayed malignancies were avoided, or that oncologic safety was preserved.

The study nevertheless has several strengths. All consecutive women with an adnexal mass presenting to the dedicated ultrasound clinic during the predefined periods were evaluated by the same highly experienced IOTA-certified examiner using the same high-end ultrasound platform and standardized terminology. ADNEX was calculated prospectively in routine care, and all operated cases included in the outcome analyses had histological verification. The pooled regression was restricted to a parsimonious model to avoid redundancy between age and the age-containing ADNEX score, and the statistical analyses were revised to use patient-level outcomes and non-parametric comparisons appropriate to the observed distributions.

Although telemedicine was not measured in this cohort and no conclusions about its effectiveness can be drawn from our data, future service models could explore remote clinical follow-up combined with prompt in-person expert ultrasonography for selected patients [20,21]. More broadly, prospective multicenter studies with comparable observation periods, complete longitudinal follow-up of conservatively managed lesions, and prespecified decision rules are needed to determine whether structured ultrasound-based risk stratification can safely improve prioritization when surgical resources are constrained.

## 5. Conclusions

In conclusion, the COVID-19 restriction period was associated with a lower proportion of evaluated women proceeding to surgery and with a different surgically treated case mix characterized by older age, higher ADNEX risk estimates, and a greater proportion of borderline or malignant histology. These findings describe risk enrichment among patients reaching surgery under constrained healthcare conditions, but they do not establish that ADNEX itself caused the change in surgical selection or that deferred patients were managed safely. Prospective studies with complete follow-up of both operated and conservatively managed women are required to determine the effectiveness and safety of ultrasound-based prioritization strategies.

## Figures and Tables

**Table 1:** Patient-level characteristics of the operated study cohorts. Values are median (IQR).

	COVID (N = 40)	Non-COVID (N = 144)	*p* Value	Rank-Biserial r
**Age**	52.0 (40.3–62.0)	39.0 (30.0–55.0)	0.0053	0.289
**ADNEX**	13.7 (4.6–54.6)	4.4 (2.2–18.6)	0.0010	0.341
**Max. lesion diameter (mm)**	74.0 (51.8–107.8)	66.0 (50.5–86.3)	0.1452	0.151

**Table 2:** Ultrasound characteristics and histological outcomes. Color score and acoustic shadowing are summarized at patient level; morphology is presented descriptively at lesion level because bilateral/multiple lesions were retained.

		Non-COVID	COVID
**Maximum Color score** *	1	88 (62.0%)	12 (30.8%)
	2	19 (13.4%)	8 (20.5%)
	3	24 (16.9%)	7 (30.8%)
***p = 0.00025***	4	11 (7.7%)	12 (17.9%)
**Lesion morphology** †	Unilocular	64 (40.3%)	10 (22.2%)
	Unilocular-solid	15 (9.4%)	4 (8.9%)
	Multilocular	32 (20.1%)	6 (13.3%)
	Multilocular-solid	23 (14.5%)	14 (31.1%)
	Solid	25 (15.7%)	11 (24.4%)
**Acoustic shadow** ‡	Present	63 (43.45%)	24 (60.00%)
***p = 0.0758***	Absent	81 (56.55%)	16 (40.00%)
**Histological outcome** §	Benign	125 (86.8%)	27 (67.5%)
***p = 0.0083***	Borderline	1 (0.7%)	5 (12.5%)
	Malignant	18 (12.5%)	8 (20.0%)

* Available color scores: non-COVID n = 142, COVID n = 39; *p*-value from chi-square test (Cramer’s V = 0.326). † Lesion-level denominators: non-COVID n = 159 lesions and COVID n = 45 lesions; no inferential *p*-value is shown because multiple lesions within a patient are not independent. ‡ Patient-level presence if an acoustic shadow was recorded in any lesion; Fisher’s exact test. § Primary comparison combined borderline and malignant outcomes versus benign histology; Fisher’s exact *p* = 0.0083; OR for non-benign histology in the COVID versus non-COVID surgical cohort 3.17 (95% CI 1.40–7.18).

**Table 3:** Exploratory pooled multivariable logistic regression for borderline/malignant versus benign histology among operated patients.

Predictor	OR (95% CI)	*p*-Value
ADNEX score (per 1 percentage point)	1.045 (1.030–1.061)	3.1 × 10^−9^
COVID period vs. non-COVID period	2.87 (1.06–7.78)	0.0383

## Data Availability

In accordance with the journal’s guidelines, the data presented in this study are available upon request from the corresponding author for the reproducibility of this study if such is requested.

## Supplementary Materials

The following supporting information can be downloaded at: https://www.mdpi.com/article/10.3390/diagnostics16172719/s1, Table S1. Histopathological spectrum of the operated cohorts based on the original routine pathology reports.
