# Supplementary material for: Changes in Ultrasound-Based Risk Stratification and Surgical Selection of Adnexal Masses During the COVID-19 Pandemic: A Single-Center Retrospective Study
Source: Diagnostics (Basel). 2026 Aug 26;16(17):2719. doi: 10.3390/diagnostics16172719 (PMC13565541; doi:10.3390/diagnostics16172719)
Supplement: Supplementary file 1 [file diagnostics-16-02719-s001.zip › diagnostics-4371810-supplementary.pdf]

Supplementary Table

S1. Histopathological Spectrum of the Operated Cohorts

Diagnostic categories were normalized for reporting from the original routine pathology report terminology. No centralized retrospective slide review was performed. Percentages use the total operated analytic cohort as denominator (non-COVID n=144; COVID n=40). Tumor grade and FIGO stage were not systematically captured and are therefore not tabulated.

**Broad category**	**Normalized histopathological category**	**Non-COVID, n (%)**	**COVID, n (%)**
Benign	Benign serous epithelial lesion	31 (21.5%)	11 (27.5%)
Benign	Endometriosis/endometrioma	33 (22.9%)	5 (12.5%)
Benign	Mature teratoma/dermoid cyst	29 (20.1%)	0 (0.0%)
Benign	Fibroma/thecoma/thecofibroma	9 (6.2%)	3 (7.5%)
Benign	Benign mucinous epithelial lesion	7 (4.9%)	2 (5.0%)
Benign	Functional/simple/hemorrhagic cyst	7 (4.9%)	1 (2.5%)
Benign	Paratubal/parovarian cyst	3 (2.1%)	2 (5.0%)
Benign	Inflammatory/tubal lesion	3 (2.1%)	0 (0.0%)
Benign	Brenner tumor	1 (0.7%)	0 (0.0%)
Benign	Struma ovarii	1 (0.7%)	1 (2.5%)
Benign	Leiomyoma	1 (0.7%)	0 (0.0%)
Benign	Ectopic pregnancy	0 (0.0%)	1 (2.5%)
Benign	Torsion/infarction/necrosis	0 (0.0%)	1 (2.5%)
Borderline	Serous borderline tumor	1 (0.7%)	3 (7.5%)
Borderline	Mucinous borderline tumor	0 (0.0%)	2 (5.0%)
Malignant	Serous carcinoma	8 (5.6%)	2 (5.0%)
Malignant	Mucinous carcinoma	1 (0.7%)	2 (5.0%)
Malignant	Adenocarcinoma/cystadenocarcinoma, unspecified	5 (3.5%)	0 (0.0%)
Malignant	Endometrial adenocarcinoma	1 (0.7%)	0 (0.0%)
Malignant	Endometrioid carcinoma	0 (0.0%)	1 (2.5%)
Malignant	Granulosa cell tumor	1 (0.7%)	0 (0.0%)
Malignant	Malignant mixed Müllerian tumor (MMMT)	0 (0.0%)	1 (2.5%)
Malignant	Leiomyosarcoma	0 (0.0%)	1 (2.5%)
Malignant	Metastatic malignancy	2 (1.4%)	1 (2.5%)
**Total**		**144 (100%)**	**40 (100%)**
